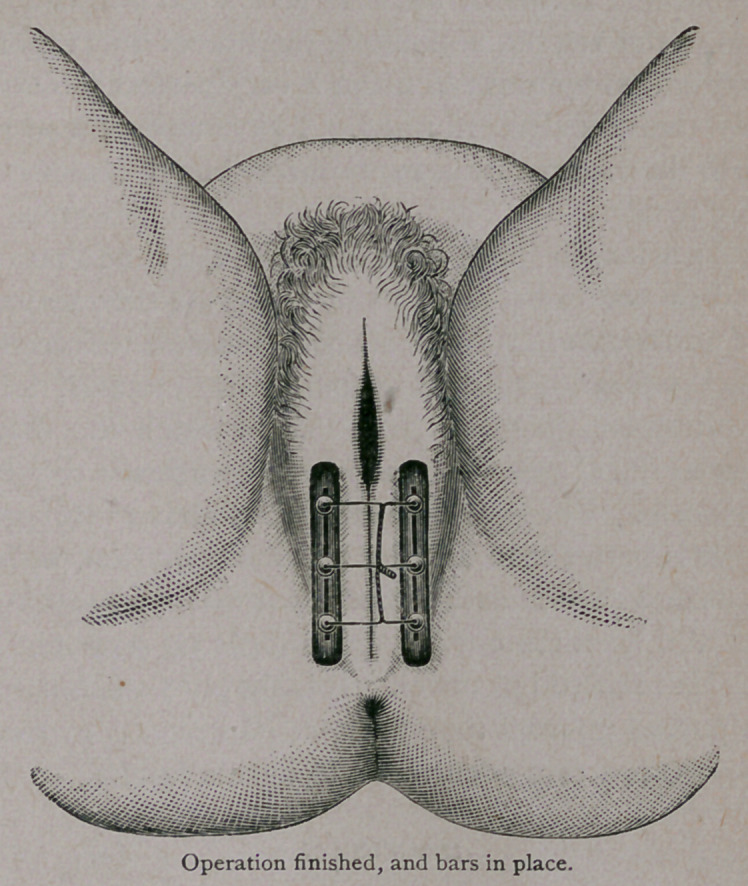# Obstetrical Society of Philadelphia

**Published:** 1889-11

**Authors:** 


### OBSTETRICAL SOCIETY OF PHILADELPHIA.

SEPTEMBER 5, 1889.

Dr. JOHN C. DA COSTA in the chair.

Dr. JOHN C. DA COSTA: An Easy Method of Repairing the Perineum.

There is probably not any operation in gynecology which gives a woman so much relief as the proper restoration of a torn perineum.

In describing this operation, I shall not say a word in regard to the anatomy of the perineum, which is the same as it was a hundred years ago. The same muscles are torn now as were torn then. This subject —tear of the perineum—may seem to be a very simple matter; but when we consider that twenty per cent. of women have their perinea torn in first labors, and four per cent. in subsequent labors, it ceases to be a little matter, and becomes one of importance.

I do not claim anything new. The operation is the result of a combination of old ideas. It is an easy and simple method of repairing the perineum, and answers equally well whether the tear is long or short. I thought I had something new in the use of these rubber bars, when I got it up eight years ago, but afterwards found that one of my ideas had been anticipated twenty years ago.

Mr. Lane, of London, in 1860, used ivory bars with small perforations, and reports thirty consecutive cases without a failure. Dr. Thompson, of Washington, used flat rubber bars with small holes in them, and reports fifty-three consecutive cases, all cured. Dr. Thomas, after speaking of the quill suture, leads us to infer that he used perforated bars, and states that he does not recall a failure in the operation.

I do not know how many present are believers in the idea advanced four or five years ago, at the meeting of the American Gynecological Society in this city, "that there is no such thing as a perineum;" but there certainly is a triangular body between the vagina on one side and the rectum on the other; and this triangular body is often torn through during labor, and becomes what I call a ruptured perineum. There are many ways of repairing it. Some are very simple, some are very striking, but very useless; what I strive to do is to restore the perineum very much as nature made it. The operation is easy, and the armamentarium is simple. We require a pair of scissors (I use a pair of blunt-pointed scissors), a perineal needle, a little silver wire and shot, a shot compressor, and two bars shaped like the cut.

Bar, ½ size.

The operation is begun at the bottom of the tear in the vagina. With one or two fingers in the rectum, I make a little slit at the lowest point, and denude subcutaneously all the tissue that has been torn. I do not know how far up I go—it may be two inches, or even nearly the length of the finger. This depends altogether upon the extent of the tear. The important thing is to get rid of all the scar tissue. Unless this is done, good union will not be secured. After denuding up the proper distance, the scissors are turned to the

Surface denuded, and stitches in place.

right and to the left, and each side denuded. Then, with four cuts of the scissors, the loosened cicatricial tissue is removed. A denudation of this kind freshens the torn perineum as, I think, no other method does. The first stitch, near the bottom of raw surface, is passed three-fourths of an inch from the edge of the cut portion, buried in the tissue the whole distance, and comes out at the same distance on the other side. The needle is then threaded with silver wire and withdrawn. The second stitch is put in in the same way. The third stitch is started in the skin like the others, and three-fourths of an inch from the edge of the cut, carried along just under the edge of the denudation the whole way around. This is the most important stitch of all. It was the idea of the late Albert H. Smith, when one of the physicians-in-chief at the Nurses' Home some years ago. The

Operation finished, and bars in place.

stitches are buried throughout, and only three are used in the operation. All that is necessary is to bring them out in nearly a straight line.

The wires are then slipped through slotted rubber bars, on each side, and shot clamped on them. After the shot are clamped, the ends of the wires are twisted over the median line, and the ends passed through a piece of catheter. In twenty-four hours there is swelling and a certain amount of inflammation. I then cut the wires off close above the shot, and this at once relieves the tension and the pain. Any desired dressing may then be applied, if it is thought advisable to use one.

What are the advantages of this operation? In the first place, you have but three stitches. I think that probably every gentleman has seen perinea operated on where there has been deep quilting, and have seen the tissue slough out because the circulation has been so interfered with that nutrition could not be maintained. These three sutures interfere very little with the circulation, and they hold together the deep parts of the wound, which is very important. When inflammation takes place, you cut the wires over the shot, the bars spread and relieve the tension and prevent any tendency to sloughing, while still supporting the parts.

After the wound is closed, you may take a piece of catgut and whip up the edges in the vagina, and along the line of the raphè. This is not necessary unless we want to make a very perfect job. The operation is easily and quickly performed. I have never timed myself, and never tried to do the operation in a hurry, but I accidentally found out how long it takes.

On one occasion, in thirty minutes from the time that I began, I had operated on two cases, and this included the time necessary to put one patient under ether from perfect consciousness to unconsciousness. The denudation is accomplished in four or five minutes.

This is a different operation from that in which the denudation is made in curved lines, and where another operation is required for any existing rectocele. The operation described above will include also a rectocele. It is better than another popular operation, which does not restore the triangle which nature made, but makes a beautiful skin-flap, which looks well from the outside, but affords no support.

I do not claim anything novel. It is simply a combination of ideas that I have picked up from time to time. In regard to the results of the operation, it is a rare occurrence to have a failure.

### DISCUSSION.

Dr. J. Price: There are a few points about which I should like to speak in connection with this and like procedures. As Dr. Da Costa has said, this is an old operation, and is illustrated in all the books. It is the old operation upon the posterior wall, and has the merit (?) he referred to, of, in many cases, making a superficial or skin perineum. The principle of suturing described is one not adopted in any other branch of surgery, and Dr. Da Costa would himself not apply this principle in any other portion of the body. He says that sometimes he denudes a distance of three inches. In no other part would he approximate such a surface with three sutures, and three sutures will not close it.

A word in regard to the denudation. He speaks of four clips of the scissors,—the button-hole, the central, and the two lateral. In many cases it is impossible to make such a denudation. You will button-hole the flap many times. That was the trouble with the Smith and Jenks operation. It is difficult to make a clean denudation in the midst of scar tissue by such a method.

One of these illustrations shows what takes place in many perineal tears. The skin-perineum side is not harmed; but if you place your finger in the sulcus on one side, you will find a sense of resistance which is absent on the other side. The sulcus is a deep one, and is a lateral tear. As has been remarked by Dr. Deaver, "It is for all the world like the lateral cut for stone." In such a case, the procedure is almost a unilateral one to bring up the pelvic floor. It is just such a state of affairs that Emmet had in view in his classical operation for the restoration of the pelvic floor or diaphragm, and he has most beautifully succeeded.

In regard to the use of this needle. Dr. Da Costa has referred to the fifty-three cases reported by Dr. Thompson of the Columbia Hospital; but he lost one or two from tetanus, and this bayonet was at the bottom of the tetanus. I look upon this needle as wholly unjustifiable in any surgery. No man has a right to have such a thing among his instruments. I am surprised that more do not die from such a stab, including, as it does, incongruous masses of tissue, skin, fat, muscles, vessels, and nerves. I remember, while a student, of seeing a death from such a stab. I use the smallest sewing-needle possible.

These procedures are very old, and are illustrated in all the old works. I consider all two-or-three-stitch methods of closing the perineum as emphatically imperfect procedures.

Dr. John C. Da Costa: What Dr. Price has said in regard to one of these illustrations has nothing to do with the subject under discussion. He refers to a tear of the vagina, which has nothing to do with a tear of the perineum. If there is a line of cicatricial tissue on one side, we do not need to denude both sides to repair the condition. It is a simple matter to remove the scar tissue and sew it up, as in any other surgical operation.

I am sorry to hear this tirade against this needle. Some very able men use this needle, and they get very good results. Albert H. Smith, who did a good deal of gynecological work, used a needle much like this. One of the most successful abdominal surgeons in Philadelphia uses a needle much like this. Surgeons in all branches of surgery use needles very like this, — either a little more or a little less curved. One who came from Europe, a year ago, showed me a long, curved needle which he brought with him and said was Tait's needle. It was precisely similar to one which I have had in my box for some years for use in complete laceration of the perineum. This is only the Baker Brown needle modified.

I do not know that Dr. Price has said anything against this operation. He has talked a good deal about the needle, and about a tear that does not apply at all. I can only say, that, despite his fears, the operations are almost uniformly successful. Any one who can do the ordinary quill operation can do this. After analyzing the various operations eight years ago, I found that the best results were obtained by the old-fashioned operation. The quill operation, however, made a V-shaped sinus to the bottom of the wound, and sometimes caused a great deal of trouble; and it was to overcome this objection that I substituted the hard rubber bars with the wires running through.

## Figures and Tables

**Figure f1:**
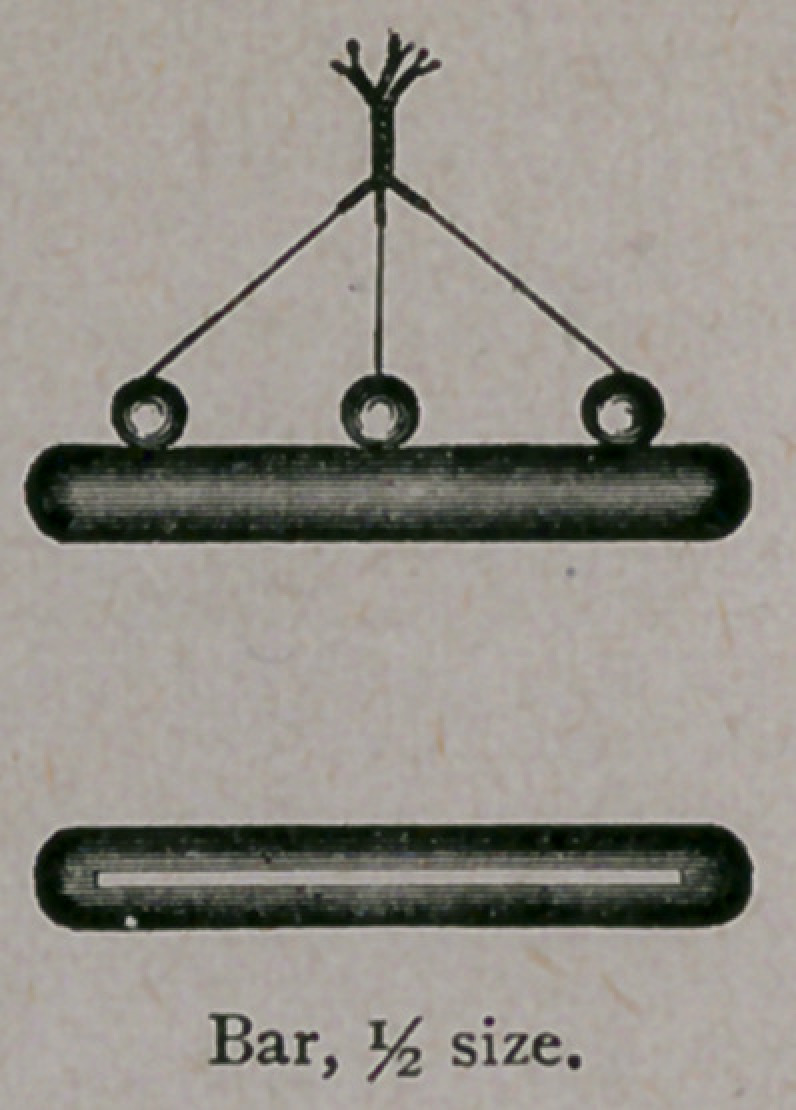


**Figure f2:**
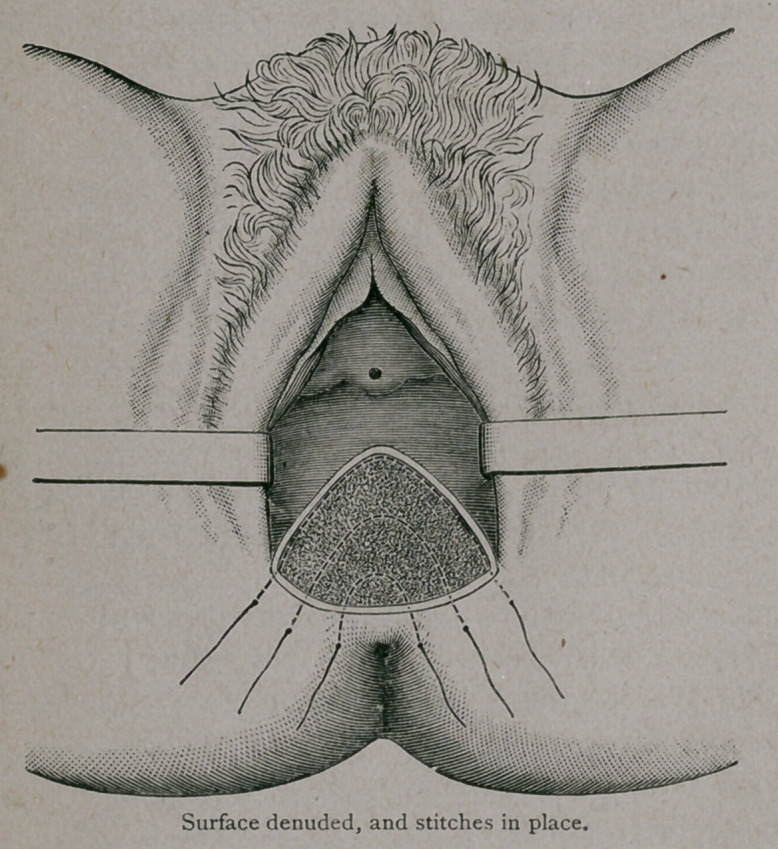


**Figure f3:**